# Antiangiogenic Drug-Induced Proteinuria as a Prognostic Factor in Metastatic Colorectal Cancer

**DOI:** 10.3390/curroncol29060319

**Published:** 2022-05-31

**Authors:** Diana Cornelia Moisuc, Mihai Vasile Marinca, Bogdan Gafton, Teodora Alexa-Stratulat, Mariana Pavel-Tanasa, Petru Cianga

**Affiliations:** 1 Immunology Department, “Grigore T. Popa” University of Medicine and Pharmacy, 700115 Iasi, Romania; cornelia-diana-i-miron@d.umfiasi.ro (D.C.M.); mariana.pavel-tanasa@umfiasi.ro (M.P.-T.); 2 Oncology Department, “Grigore T. Popa” University of Medicine and Pharmacy, 700115 Iasi, Romania; mihai.marinca@umfiasi.ro (M.V.M.); bogdan.gafton@umfiasi.ro (B.G.); teodora.alexa-stratulat@umfiasi.ro (T.A.-S.); 3 Oncology Department, Regional Institute of Oncology, 700483 Iasi, Romania; 4 Immunology Department, “St. Spiridon” Hospital, 700111 Iasi, Romania

**Keywords:** bevacizumab, chemotherapy, metastatic colorectal cancer, proteinuria, prognostic factor

## Abstract

Treatment with bevacizumab is known to cause adverse events such as proteinuria and hypertension, amongst others. However, while bevacizumab-induced hypertension has been linked to increased overall survival (OS), data on proteinuria are controversial. We performed a retrospective analysis to observe the influence of adverse events developed during treatment with bevacizumab and chemotherapy on the OS in patients with metastatic colorectal cancer (mCRC). Kaplan–Meier and log-rank analyses were used to assess differences in OS, and hazard ratios (HR) were estimated using Cox models. Out of the 3497 mCRC patients admitted to our center between 2014 and 2019, 150 met the criteria for inclusion in our analysis. Out of these, 50.7% experienced proteinuria and had reached a longer OS (40 versus 25 months, *p* = 0.015) and progression-free survival (15 versus 12 months, *p* = 0.039). The following groups were identified as having a lower risk of death: patients with proteinuria (HR 0.589; 95% CI 0.402–0.863; *p* = 0.007), one metastatic site (HR 0.533; 95% CI 0.363–0.783; *p* = 0.001), and non-metastatic stage at diagnosis (HR 0.459; 95% CI 0.293–0.720; *p* = 0.001). Patients with anemia and diabetes had an increased risk of death. Proteinuria emerges as a useful prognostic factor in mCRC patients undergoing bevacizumab-based systemic therapy, and it could be easily integrated into the decision-making process, thus allowing physicians to further individualize systemic treatments.

## 1. Introduction

Colorectal cancer (CRC) has an increased incidence in both men and women [1]. If diagnosed at an early stage, it is associated with a good prognosis [2]. However, 20–25% of patients already have metastases at the time of diagnosis and about half of those diagnosed at an early stage will eventually develop metastatic disease [3]. Surgery and fluoropyrimidine-based chemotherapy continue to represent the treatment backbone of CRC, but the advent of molecular-targeted therapies has changed the treatment landscape and greatly influenced the prognosis of metastatic disease over the last 15 years [4].

One of the major targets of the biological therapies is the cell proliferation pathway, which in CRC depends on Epidermal Growth Factor Receptor signaling. Monoclonal antibodies such as cetuximab or panitumumab have been successfully used, in conjunction with chemotherapy, for the treatment of patients not harboring mutations in the RAS oncogenes (i.e., wild-type KRAS and NRAS). Moreover, the BRAF mutations such as V600E or V600K have shown prognostic but not predictive significance for this group of patients in various studies [5,6].

Angiogenesis has an important role in tumor proliferation and metastasis. Vascular Endothelial Growth Factor (VEGF) is a key mediator of this process, and, as such, it is also a major target for many biological therapies. Inhibition of angiogenesis has become the standard approach in certain types of cancers such as colorectal, bronchopulmonary, ovarian, renal, breast, and cervical cancer [7,8,9]. However, despite extensive research, one of the major drawbacks of antiangiogenic therapies continues to be the lack of predictive biomarkers.

A current global issue is the cost of anticancer drugs, for which more than USD 100 billion is spent annually worldwide [10]. The cost-effectiveness ratio of bevacizumab for mCRC is USD 571.240 per quality-adjusted life years in the first-line setting [11]. Identifying a prognostic or predictive marker for bevacizumab therapy would help individualize treatment and alleviate the burden of increased cost.

In combination with chemotherapy, bevacizumab (a humanized IgG monoclonal antibody that binds to VEGF-A and prevents activation of the tyrosine kinase domain of its receptors VEGFR1 and VEGFR2) has been shown to be effective in clinical trials by increasing overall survival (OS), progression-free survival (PFS), and response rate (RR) [8,12,13,14,15,16]. However, adverse events (AEs) of bevacizumab, in addition to those induced by chemotherapy, may negatively impact treatment outcomes. Hematological, digestive, and neurological toxicity have been reported in patients with CRC treated with chemotherapy [17,18,19]. Bevacizumab is also associated with several particular side events such as high blood pressure, risk of bleeding, proteinuria, fistulas, gastrointestinal perforations, thromboembolic events, impaired wound healing, and heart failure [12,20].

Bevacizumab was associated with the onset of proteinuria in 10 to 30% of the patients with CRC and up to 71% of the patients with renal cancer [21]. Although studies have shown a relationship between bevacizumab and the risk of developing proteinuria [22,23,24], the mechanism by which it occurs is not yet fully understood. Most of the time, AEs are reported in clinical trials to verify the treatment safety and not to evaluate their influence on OS. Studies have shown that the occurrence of proteinuria can be considered a predictive [25] or prognostic factor [26], but others have failed to demonstrate this relationship [21,27].

Several studies reported that febrile neutropenia requires a dose reduction of chemotherapy, leading further to decreased OS in cancer patients [28,29]. Anemia is frequently observed in CRC patients due to tumor bleeding, especially in rectal cancer. In patients with squamous cell carcinoma of the anal canal and anal margin, for example, hemoglobin concentration was an independent prognostic factor for OS, with anemic patients having a poor prognosis [30]. Several studies investigated the impact of preoperative anemia in CRC patients [31,32], but, to the best of our knowledge, there are no data regarding the impact of myelosuppression induced anemia or other adverse events of chemotherapy and bevacizumab.

The aim of this retrospective study was to analyze the influence of proteinuria, hematological, hepatic, renal, digestive, and neurological toxicity on the results of treatment with bevacizumab plus chemotherapy in patients with mCRC. Identifying a biomarker may help to select the mCRC patient’s subgroup that will have a favorable outcome following treatment with bevacizumab and chemotherapy.

## 2. Materials and Methods

### 2.1. Patients

We performed a retrospective analysis of patients diagnosed with mCRC treated with bevacizumab and chemotherapy in our center. Inclusion criteria were: age over 18 years, histologically confirmed colorectal cancer, first-line bevacizumab treatment, adequate baseline hematological, hepatic and renal function, and performed urinalysis before and during treatment. Patients with incomplete data were excluded.

Patients received bevacizumab 7.5 mg/kg every 3 weeks or 5 mg/kg every 2 weeks along with standard dose chemotherapy regimens: CapeOX (oxaliplatin 130 mg/m^2^ iv and capecitabine 1000 mg/m^2^ twice a day oral, day 1–14), mFOLFOX 6 (oxaliplatin 85 mg/m^2^ iv, fluorouracil 400 mg/m^2^ bolus and 2400 mg/m^2^ iv 46 h and leucovorin 400 mg/m^2^ iv), CapeIRI (irinotecan 240 mg/m^2^ iv and capecitabine 1000 mg/m^2^ twice a day oral, day 1–14), FOLFIRI (irinotecan 180 mg/m^2^ iv, fluorouracil 400 mg/m^2^ bolus and 2400 mg/m^2^ iv 46 h and leucovorin 400 mg/m^2^ iv), de Gramont (fluorouracil 400 mg/m^2^ bolus and 2400 mg/m^2^ iv 46 h and leucovorin 200 mg/m^2^ iv), or capecitabine monotherapy.

For each case, several types of data were collected by reviewing patients’ medical records: demographic characteristics, types of chemotherapy, pre-existing comorbidities, treatment-related AEs (including the onset of proteinuria), PFS, and OS. Hematological (anemia, neutropenia, thrombocytopenia), hepatic, and renal toxicity were classified according to Common Terminology Criteria for Adverse Events (CTCAE) v4.0 by analysis of complete blood count (CBC), differential liver function (GGT, gamma-glutamyl transpeptidase; ASAT, aspartate-aminotransferase; ALAT, alanine-aminotransferase), and creatinine. Proteinuria was assessed in the summary urine test and was noted to be present or absent, with a cut-off level of 30 mg/dL. Tumor response was evaluated after at least 6 months of treatment and interpreted according to the Response Evaluation Criteria in Solid Tumors (RECIST) v1.1 provisions [33]: complete response (CR, disappearance of all lesions), partial response (PR, at least a 30% decrease in the sum of diameters of target lesions), stationary disease (SD, decrease by less than 30% or increase by less than 20%), progressive disease (PD, at least a 20% increase in the sum of diameters of target lesions or the occurrence of new lesions).

### 2.2. Statistical Analysis

For statistical analysis, we used the SPSS v.16.0 software (SPSS Inc., Chicago, IL, USA). The qualitative and quantitative variables were characterized by frequency, mean, median, and standard deviation to describe the basic characteristics of the studied population. The Chi-square test and Wilcoxon rank-sum test were used to compare median values and proportions. The Kaplan–Meier curve was used to estimate PFS and OS, and the log-rank test was used to compare groups, with a *p*-value of <0.05 indicating statistical significance. A logistic regression analysis was performed using the development of proteinuria as the dependent variable and the following factors as independent variables: previous hypertension, diabetes, other cardiovascular comorbidities, age, gender, and first-line chemotherapy regimen. To identify toxicities influencing OS, a univariate analysis was performed, and statistically significant factors were included in the multivariate Cox analysis using OS as the dependent variable. Other independent variables included were proteinuria, anemia, age groups (less or more than 65 years), comorbidities, stage at diagnosis (metastatic versus non-metastatic), number of metastatic sites (one versus more than one), and tumor location (left versus right). Furthermore, in order to minimize the case selection bias within the two groups (patients with and without proteinuria), propensity score matching was also used (XLSTAT v.2022). Stratification was performed using the following confounders: age, gender, pre-existing hypertension, other cardiovascular comorbidities, diabetes, tumor location, stage at diagnosis, primary tumor resection, number of metastatic sites, chemotherapy regimen, and tumor response, as listed in Table 1.

## 3. Results

### 3.1. Baseline Patient Disposition and Disease Characteristics

**Figure 1:**
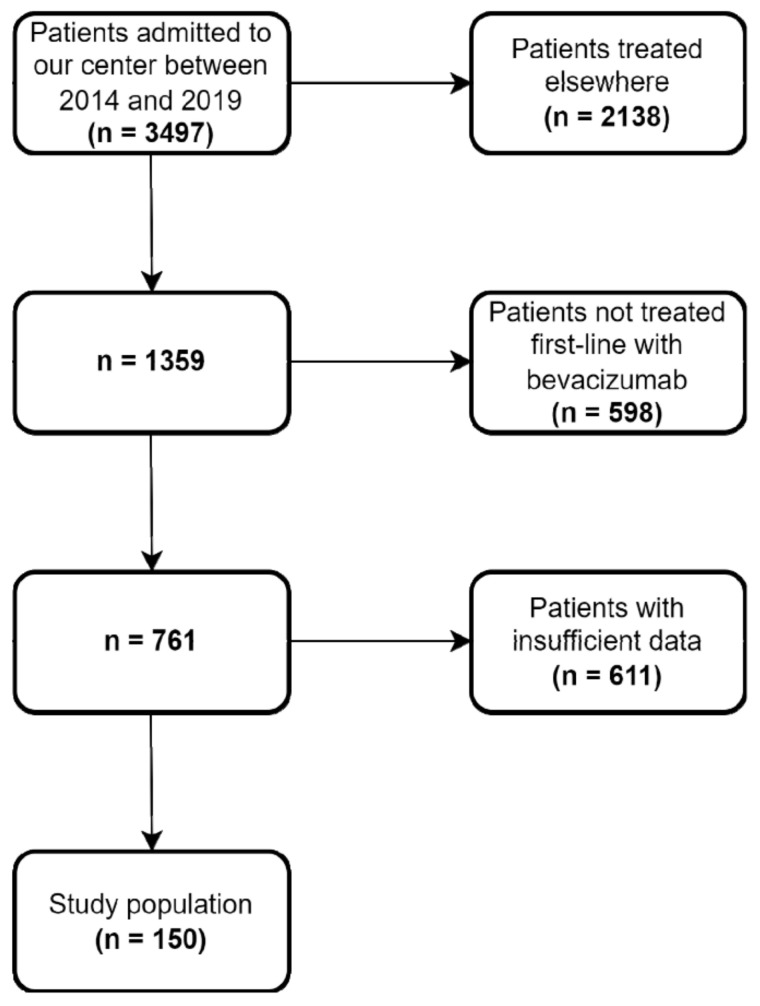
Flowchart detailing patient selection for the study.

A total of 150 mCRC patients undergoing first-line chemotherapy concomitant with bevacizumab between 2014 and 2019 were included in the analysis (Figure 1). The median age of the patients was 64 ± 9.6 years. Most of the tumors (67%) were located on the descending colon. Mutations in the RAS (KRAS, NRAS) and BRAF (V600E) genes were present in 60 patients out of the 107 for whom these data were available. The most common site for metastasis was the liver (63%), followed by the lung (17%) and bone (5%), while 51 patients presented more than one site of metastasis. The median follow up was 27 months. Baseline patient disposition and disease characteristics according to proteinuria are summarized in Table 1. There were no significant differences between groups in terms of gender (*p* = 0.25), age (*p* = 0.28), cardiovascular comorbidities (*p* = 0.58), diabetes (*p* = 0.47), primary tumor location (*p* = 0.44), or associated chemotherapy regimen (*p* = 0.97).

### 3.2. Adverse Events

We analyzed both bevacizumab- and chemotherapy-related toxicities. The most common adverse events and the incidence of grade 3 or higher AEs during the treatment period are shown in Table 2. Hepatic toxicity and anemia were the most common AEs of any grade; hepatic toxicity and neutropenia were the most common grade 3 and 4 AEs. Grade 3 or higher oxaliplatin-related neurological toxicity (peripheral neuropathy) occurred in 11 patients.

#### 3.2.1. Proteinuria

**Figure 2:**
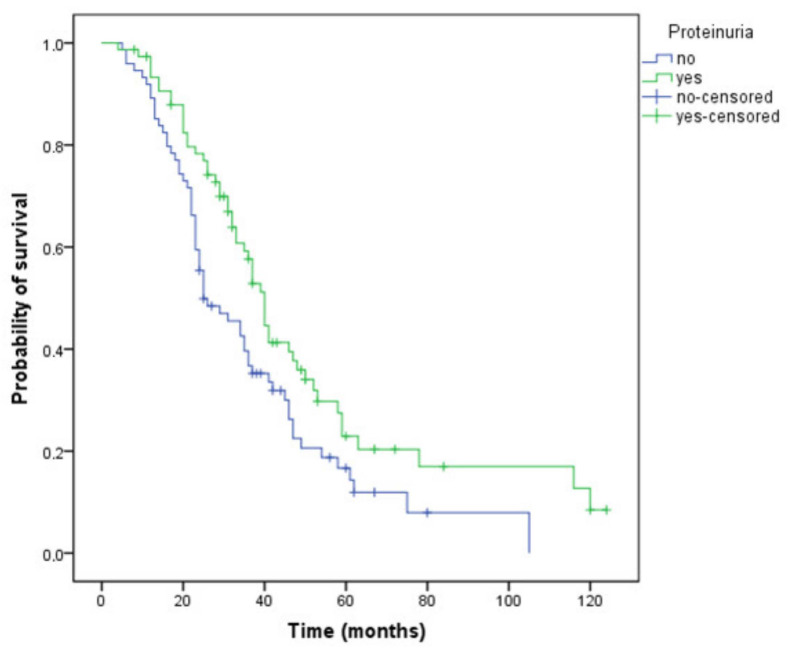
Kaplan–Meier curve of overall survival for patients who have or have not developed proteinuria during treatment (OS, 40 versus 25 months, *p* = 0.015).

Proteinuria was present in 50.7% of patients. The median time to the onset of the proteinuria was 10 (range 1–32) months. None of the factors analyzed using the logistic regression method were related to the development of proteinuria: pre-existing hypertension (*p* = 0.08), presence of diabetes (*p* = 0.477), other cardiovascular comorbidities (*p* = 0.589), gender (*p* = 0.259), age (*p* = 0.383), or chemotherapy regimen (oxaliplatin-based, *p* = 0.965; irinotecan-based, *p* = 0.835; fluorouracil/capecitabine-based, *p* = 0.976). Median PFS was 13 months (95% CI 11.9–14.0) in the entire study population, and median OS was 35 months (95% CI 30.9–39.0). Patients who developed proteinuria during treatment had a longer PFS (15 versus 12 months, *p* = 0.039) and OS (40 versus 25 months, *p* = 0.015) compared with those without proteinuria (Figure 2). The disease control rate (DCR) was also higher in patients with proteinuria (76.3% versus 68.9%), but the difference was not statistically significant (*p* = 0.309).

#### 3.2.2. Anemia

**Figure 3:**
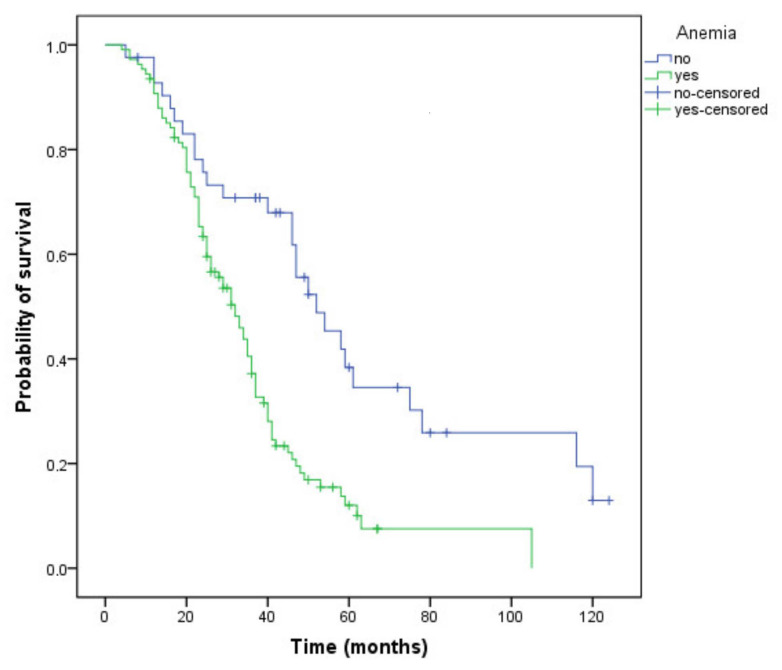
Kaplan–Meier curve of overall survival for patients who have or have not developed anemia during treatment (OS, 32 versus 52 months, *p* < 0.001).

Patients who had anemia during treatment, regardless of grade, had a 20-month shorter survival (Figure 3) compared with those not experiencing this AE (32 versus 52 months, *p* < 0.001). The DCR was higher in patients without anemia (73.8% versus 72.2%), but the difference did not reach statistical significance (*p* = 0.84).

### 3.3. Disease Control Achievement and Stage at Diagnosis

**Figure 4:**
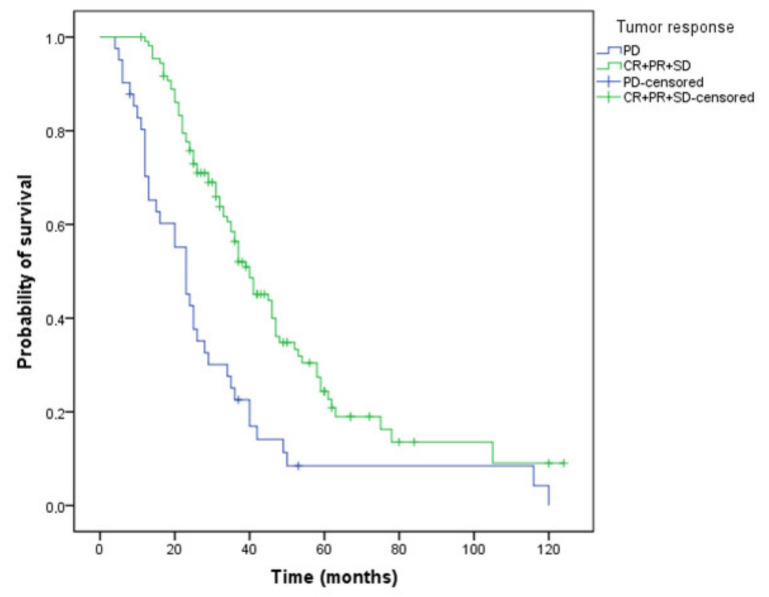
Kaplan–Meier curve of overall survival for patients who have or have not obtained a tumor response (OS, 40 versus 23 months, *p* < 0.001, PD—progressive disease, CR—complete response, PR- partial response, SD—stabile disease).

Patients who achieved disease control with first-line chemotherapy plus bevacizumab treatment had a significantly longer survival: 40 versus 23 months (Figure 4) compared to those with progressive disease (*p* < 0.001).

**Figure 5:**
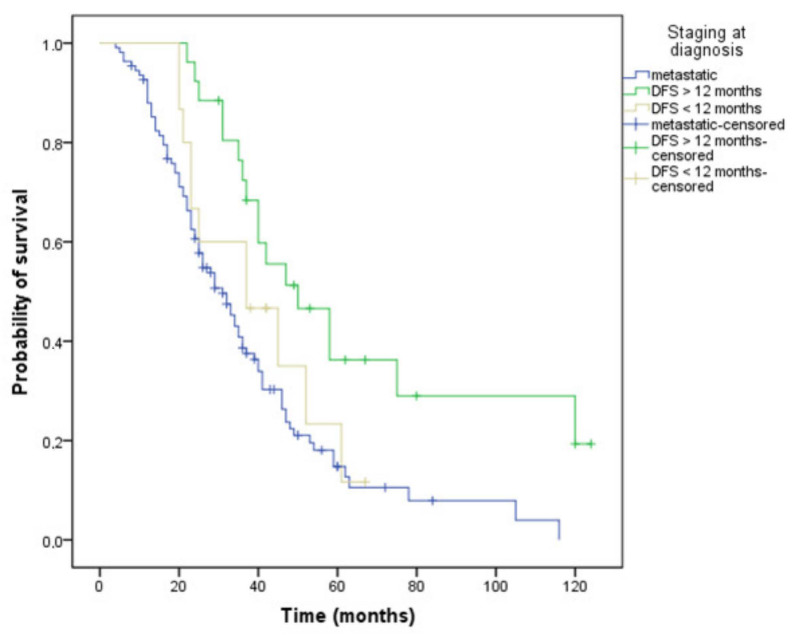
Kaplan–Meier curve of overall survival depending on the stage at diagnosis: metastatic or non-metastatic: DFS (disease-free survival) less or more than 12 months (OS, 31 versus 37 versus 50 months, *p* = 0.002).

Patients with the metastatic stage at diagnosis had a 31-month OS. Survival of those who had progressed in less than 12 months after completion of adjuvant chemotherapy was 37 months, while patients progressing after more than 12 months from completion of adjuvant treatment achieved the best OS, 50 months (*p* = 0.002) (Figure 5). Patients with a single metastatic site, regardless of location, had better survival rates compared to patients with at least two metastatic sites (39 versus 29 months, *p* = 0.017).

### 3.4. Prognostic Factors

The following two toxicities were significantly associated with OS in the univariate analysis: proteinuria (*p* = 0.017) and anemia (*p* = 0.001). The other adverse events affected quality of life but not survival: neutropenia (*p* = 0.446), thrombocytopenia (*p* = 0.259), hepatic toxicity (*p* = 0.169), renal toxicity (*p* = 0.164), neurological toxicity (*p* = 0.364), and digestive toxicity (*p* = 0.224).

In the multivariate analysis (Table 3), the following groups had a lower risk of death: patients with proteinuria (HR 0.589; 95% CI 0.402–0.863; *p* = 0.007), one metastatic site (HR 0.533; 95% CI 0.363–0.783; *p* = 0.001), and non-metastatic stage at diagnosis (HR 0.459; 95% CI 0.293–0.720; *p* = 0.001). Patients with anemia (HR 2.437; 95% CI 1.531–3.881; *p* < 0.001) and diabetes (HR 1.828; 95% CI 1.002–3.337; *p* = 0.049) had an increased risk of death.

### 3.5. Propensity Score Matching

**Figure 6:**
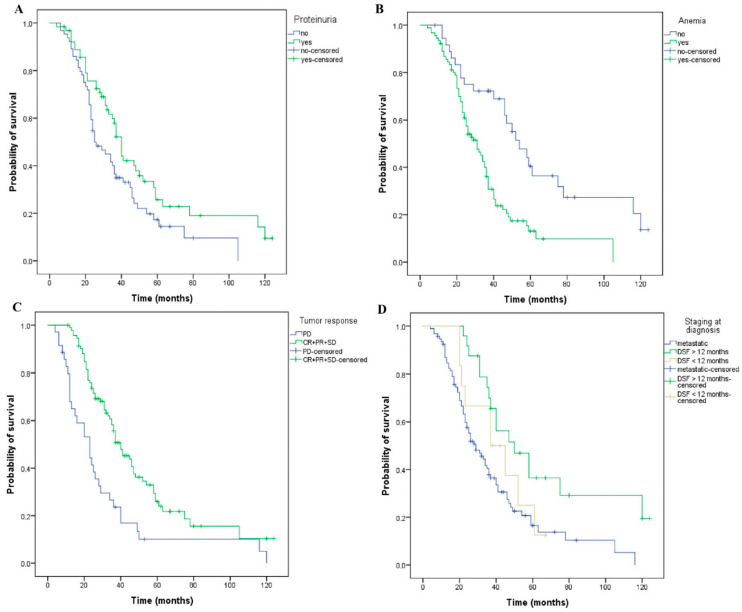
Kaplan–Meier curves of overall survival for matched patients using the propensity score matching method. The patients have or have not developed: (**A**) proteinuria (OS, 40 versus 25 months, *p* = 0.028), and (**B**) anemia (OS, 31 versus 54 months, *p* < 0.001) during treatment. (**C**) The patients have or have not obtained a tumor response (OS, 40 versus 23 months, *p* < 0.001, PD—progressive disease, CR—complete response, PR—Partial response, SD-stabile disease). (**D**) The patients had or had no metastases at the stage of diagnosis, DFS = disease-free survival less or more than 12 months (OS, 29 versus 37 versus 50 months, *p* = 0.006).

Additionally, the XLSTAT v.2022 version of the propensity score matching was employed to further minimize the case selection bias within the two groups (patients who have or have not developed proteinuria during treatment with bevacizumab). We have thus identified 64 pairs of patients, representing 85% of the total analyzed cases (Table 4 and Supplementary Table S1-PSM). In this context, patients who developed proteinuria had a longer OS (40 versus 25 months, *p* = 0.028) as compared with those without proteinuria (Figure 6A). Patients who maintained a normal hemoglobin value had a longer OS compared with anemic patients (54 versus 31 months, *p* < 0.001) (Figure 6B). Moreover, patients who achieved disease control with first-line therapy had a longer OS (40 versus 23 months, *p* < 0.001) (Figure 6C) compared to those with progressive disease. Patients with a metastatic stage at diagnosis had a 29-month OS, significantly shorter than those with a non-metastatic stage at diagnosis with DFS greater than 12 months or less than 12 months, respectively (29 versus 37 versus 50 months, *p* = 0.006) (Figure 6D). To further verify these results, we performed a multivariate Cox analysis for the above-mentioned subset of patients. All the parameters we have considered (proteinuria, anemia, diabetes, stage at diagnosis, and the number of metastatic sites) generated consistent significant results, thus strengthening our previous conclusions regarding their influence on OS (Table 5).

## 4. Discussion

Numerous studies and retrospective analyses have been performed to identify novel prognostic factors that could be readily used in the clinical setting for CRC patients. Factors such as the location of the primary tumor, histologic grade, history of primary surgery, metastasectomy, performance status, peritoneal metastases, lactate dehydrogenase, PFS interval prior to liver surgery, carcinoembryonic antigen levels, liver toxicity (transaminases), and the size of the two largest lesions on CT scans have been evaluated in several prospective and retrospective studies [34,35,36]. However, no prognostic or predictive biomarkers specific to patients undergoing antiangiogenic systemic therapy have been identified to date. Although VEGF is one of the most studied biomarkers in clinical trials [37,38,39], the data available so far are still contradictory.

The main purpose of this study was to analyze the putative relationship between the occurrence of treatment-related adverse events, specifically bevacizumab-induced proteinuria, and OS. Results showed that the category of patients who developed proteinuria had a significantly better OS and PFS compared to those who did not experience this AE.

Previous studies have shown a close correlation between the use of bevacizumab and the development of proteinuria [22,23,24]. Proteinuria has also been studied as a predictive factor, but no consensus was reached. Zee et al. reported significantly lower survival rates in patients with colorectal cancer treated with antiangiogenic therapy if they developed proteinuria grade 2 or higher, as opposed to grade 0–1 (OS 4.2 months versus 23.9 months) [40]. In another study, no correlation was found between the severity of proteinuria and survival in patients with mCRC treated with bevacizumab [21]. Feliu et al. demonstrated that the occurrence of proteinuria is correlated with the response rate. However, they included only elderly patients in the study. Patients with moderate and severe proteinuria had a response rate of 56% and an OS of 22 months compared to 37% and 20.1 months, respectively, in patients with grade 0–1 proteinuria, but the survival advantage was not statistically significant [27]. Another study showed that the early development of both hypertension and proteinuria after the initiation of bevacizumab in patients with breast cancer is associated with tumor response rate, and the authors suggested that these two side effects could be considered predictive [25].

Our results differ from the mentioned studies due in part to variations between target populations and also to differences in methods and parameter definitions. Another very important source of bias is the small patient numbers in all these studies, which we have also emphasized. For example, Zee et al. conclude that grade 2 proteinuria represents a pejorative factor for OS but not for PFS or RR in their subjects. In our slightly larger sample, we have found the presence of proteinuria to carry a better prognosis for both OS and PFS, and we hypothesized it might be regarded as a surrogate marker for higher efficacy of anti-angiogenic therapies. While qualitative analysis is definitely more error-prone, it offers a more affordable and feasible evaluation of proteinuria than quantitative methods. We only performed a qualitative evaluation (yes versus no) of proteinuria and identified it in 76 patients in our sample, while the more quantitative analysis of Zee et al. (though also based on dipstick urine protein level) found grade 1 proteinuria in 12 patients and grade 2 in only 4 of their patients, respectively. Further, they also noted that proteinuria (at any grade) has not been associated with kidney dysfunction, hence the grade 2 cut-off might indeed be considered somewhat arbitrary.

Other authors correlated the development of proteinuria with the cumulative dose of bevacizumab, the number of cycles administered [27,41], systolic blood pressure values above 130 mmHg [42], or the presence of diabetes [43]. Out of all these, only the presence of diabetes was analyzed in the present study, but neither this nor any other variable appeared to significantly influence the development of proteinuria; 10 of 17 diabetic patients included in our cohort developed proteinuria during treatment.

A meta-analysis that included data from 16 studies showed that adding bevacizumab to chemotherapy increases 4.79-fold the median risk of grade 3–4 proteinuria. This increase varied with cancer type (e.g., 2.52 for colorectal cancer; 48.7 for kidney cancer) and showed a linear relationship with the dose of bevacizumab (e.g., 2.62 at a dose of 2.5 mg/kg and 8.56 at 5 mg/kg, compared to chemotherapy alone) [22].

Several potential angiogenesis-related mechanisms have been proposed for the induction of proteinuria. As a response to hypoxia and decreased proteasomal degradation of hypoxia-inducible factor 1-alpha (HIF-1-alpha), both production of VEGF by podocytes and consecutive activation of the VEGF-2 receptor on glomerular capillary endothelial cells are increased. Conversely, VEGF/VEGFR-2 inhibition causes a loss of podocytes, endothelial fenestration, glomerulosclerosis, and tubulointerstitial fibrosis [44]. In addition, inhibition of VEGF may cause glomerular thrombotic microangiopathy and membranoproliferative changes [45].

The correlation between bevacizumab-induced toxicity and outcome may have a genetic explanation. Studies identified genetic variants of VEGF and VEGFR having potentially predictive value for antiangiogenic therapy [46,47]. Hansen et al. reported that VEGFR-1 319 C/A single nucleotide polymorphism was associated with the response rate in mCRC patients treated with bevacizumab and chemotherapy [48]. Another study suggested that genetic variants of VEGF may be linked to the risk of toxicity. Breast cancer patients treated with bevacizumab and chemotherapy carrying VEGF-634 CC and VEGF-1498 TT genotypes had a lower incidence of grade 3 or 4 hypertension [49]. Nikzamir et al. reported that VEGF + 405 GG genotype was a predictive factor for albuminuria in patients with type 2 diabetes [50]. Patients developing proteinuria during bevacizumab treatment may be carriers of such variants. However, the role of genetic variants of VEGF in the development of bevacizumab-related proteinuria has not been studied yet.

No guidelines are currently available for the management of bevacizumab-induced proteinuria, although there is general consensus on the necessity to prevent subsequent renal failure, cardiovascular complications, as well as tumor progression due to permanent discontinuation of biologic therapy if proteinuria exceeds 2 g/24 h or nephrotic syndrome occurs, respectively.

In the present study, the occurrence of at least one episode of anemia during treatment was a negative prognostic factor for OS. Survival decreased significantly according to the grade of anemia (20 months for grade 3 versus 31 months for grade 2 versus 34 months for grade 1; no grade 4 or 5 anemia was reported). This is in accord with the conclusions of a meta-analysis reporting that anemia at any point during the course of the disease increases the risk of death in cancer patients. When presenting anemia, the relative risk of death was increased by 19% in lung cancer, by 75% in head and neck carcinomas, and by 47% in prostate cancer patients [51]. Anemia during chemotherapy also affects OS by the deriving necessity to delay or reduce the dose of chemotherapy. In addition, anemia produces tumor hypoxia that reduces the effectiveness of chemotherapy and bevacizumab. Although anemia can be corrected, there is no evidence to improve long-term prognosis after performing therapeutic procedures (transfusions, stimulation of erythropoiesis).

Another factor influencing both OS and disease-free survival (DFS) is the tumor stage at diagnosis [52,53]. In multivariate analyses, we have split our study population into two groups (upfront metastatic, *n* = 109; and non-metastatic, *n* = 41) and found that initially non-metastatic patients had significantly better survival rates. Patients with a single metastatic site had a 10-month longer survival than patients with at least two metastatic sites. The present study also showed that tumor volume has a negative impact on the prognosis. Köhne et al. analyzed a panel of clinical, hematological, and biochemical factors to identify prognostic markers of CRC patients treated with fluorouracil-based chemotherapy, and the results showed that the number of metastatic sites, along with other factors, classified patients with mCRC into different risk categories. The most unfavorable risk was for patients with ECOG performance status 0–1, more than one metastatic site, and alkaline phosphatase over 300 U/L. Platelets (>400 × 10^9^/L), alkaline phosphatase level (>300 IU/L), WBC count (>10 × 10^9^/L), and hemoglobin (<11 g/dL) predicted an inferior survival probability. Lactate dehydrogenase, bilirubin, ALAT, ASAT, total protein, albumin, and carcinoembryonic antigen (CEA) levels were not significant [54]. The number of metastatic sites is considered a negative prognostic factor not only for CRC [55] but also for lung cancer [56], esogastric cancer [57], and for endometrial carcinoma [58].

Another multivariate analysis concluded that primary tumor location, performance status, number of metastatic sites, baseline CEA level, and platelets may be considered prognostic factors in patients with mCRC treated with oxaliplatin and bevacizumab [59]. In the current analysis, renal, hepatic, digestive, and neurological toxicities affected the quality of life to various degrees but did not influence OS.

Our research is subject to several limitations. We have included in our study 150 carefully chosen patients, a number that precludes definitive conclusions or recommendations based on the results above. However, our results provide additional data on the prognostic role of proteinuria and warrant more extensive prospective studies in order to validate the present findings. Another study limitation is the retrospective nature of the study and selection bias. For example, about 20% of patients present with de novo metastatic colon cancer. In the present study, the number of de novo metastatic diseases is >70%, which suggests patients with a previous diagnosis of early-stage colorectal cancer are not reflecting the source population, and the finding that previous history of an early-stage disease has a better prognosis could merely be due to selection bias. In addition, some of the known prognostic factors including BRAF status, metastasectomy, subsequent line of chemotherapy, and baseline performance status were not examined in the analysis. The study might have a potentially short follow-up bias. Studies reported a wide range time-to-onset of proteinuria, from 3 weeks to 37 months, with a median of 5.6 months from the start of bevacizumab. The median follow up for the present study was 27 months, so several additional cases of late proteinuria occurring in our population were thus not considered. It is common knowledge that hypertension influences the development of proteinuria through various mechanisms. In the logistic regression analysis, we included only pre-existing hypertension without considering whether blood pressure had been controlled by anti-hypertensive treatment. It is also possible that some patients might have developed hypertension during treatment, and this might have influenced the occurrence of proteinuria. Another study limitation is the heterogeneity of the patients included, whether synchronous or metachronous metastases, and the lack of information about the metastatic disease characteristics that may influence the carcinological results.

## 5. Conclusions

The results of our study suggest that, in addition to the non-metastatic stage at diagnosis and one metastatic site, the development of proteinuria during first-line treatment with bevacizumab and chemotherapy of patients with mCRC was an independent prognostic factor for OS and correlates with a better prognosis. Despite the fact that literature data are controversial in terms of the prognostic role of proteinuria, the results of our study argue in favor of it. The presence of diabetes, pre-existing hypertension, and other cardiovascular conditions did not increase proteinuria risk in the studied group. Neutropenia, thrombocytopenia, hepatic, renal, and neurological toxicity do not influence OS. The presence of anemia during treatment and diabetes were negative prognostic factors.

## Figures and Tables

**Table 1:** Patients’ characteristics.

Characteristic	Patients without Proteinuria (*n* = 74)		Patients with Proteinuria (*n* = 76)	
	*n*	%	*n*	%
**Median age, years (range)**	62 (33–82)		65 (39–82)	
**Gender**				
Male	39	45	47	55
Female	35	55	29	45
**Pre-existing hypertension**				
Yes	20	51	19	49
No	54	49	57	51
**Other cardiovascular comorbidities**				
Yes	13	45	16	55
No	61	50	60	50
**Diabetes**				
Yes	7	41	10	59
No	67	50	66	50
**Tumor location**				
Left colon	52	51	49	49
Right colon	22	45	27	55
**Stage at diagnosis**				
Metastatic	54	49	55	51
Non-metastatic	20	49	21	51
**Primary tumor resection**				
Yes	57	47	65	53
No	17	61	11	39
**Sites of metastasis**				
One	46	46	53	54
More than one	28	55	23	45
**Chemotherapy regimen**				
Oxaliplatin-based	53	49	54	51
Irinotecan-based	14	50	14	50
Fluorouracil/Capecitabine-based	7	47	8	53
**Tumor response**				
CR	4	50	4	50
PR	17	49	18	51
SD	30	45	36	55
PD	23	56	18	44

CR = Complete response; PR = Partial response; SD = Stabile disease; PD = Progressive disease.

**Table 2:** Adverse events of bevacizumab and chemotherapy ^1^.

Event	All Grades N (%)	Grade ≥ 3 N (%)
Any	143 (95)	57 (38)
Proteinuria	76 (50.7)	*
Anemia	108 (72)	8 (5.3)
Neutropenia	84 (56)	13 (8.7)
Thrombocytopenia	75 (50)	0
Renal toxicity	57 (38)	2 (1.3)
Hepatic toxicity	121 (80.6)	40 (26.6)
Neurological toxicity	69 (46)	11 (7.3)
Digestive toxicity ^2^	44 (29)	3 (2)

^1^ Classified according to Common Terminology Criteria for Adverse Events v4.0. ^2^ Digestive toxicity refers to nausea, vomiting, and/or diarrhea. * Evaluation of proteinuria was qualitative only.

**Table 3:** Univariate and multivariate prognostic factors for longer OS in metastatic colorectal patients treated with bevacizumab and chemotherapy.

Characteristics	Univariate Analysis			Multivariate Analysis		
	HR	95% CI	*p*-Value	HR	95% CI	*p*-Value
Proteinuria	0.635	0.437–0.923	0.017	0.589	0.402–0.863	0.007
Anemia	0.405	0.255–0.643	<0.001	2.437	1.531–3.881	<0.001
Age ^1^	1.268	0.875–1.837	0.210	-	-	-
Cardiovascular comorbidities	1.015	0.650–1.585	0.948	-	-	-
Diabetes	1.192	0.666–2.134	0.554	1.828	1.002–3.337	0.049
Staging at diagnosis ^2^	0.493	0.319–0.764	0.002	0.459	0.293–0.720	0.001
Number of metastatic sites ^3^	0.638	0.438–0.929	0.019	0.533	0.363–0.783	0.001
Tumor location ^4^	0.976	0.800–1.189	0.807	-	-	-

^1^ Less or more than 65 years; ^2^ Staging at diagnosis: metastatic versus non-metastatic; ^3^ One or more than one metastatic site; ^4^ Left versus right.

**Table 4:** Summary of the matched observations according to the presence of proteinuria.

Categories	Number	Matched	Percentages	Unmatched	Percentages
yes	76	64	84%	12	16%
no	74	64	86%	10	14%

**Table 5:** Univariate and multivariate prognostic factors for longer OS in metastatic colorectal patients treated with bevacizumab and chemotherapy after performing propensity score matching.

Characteristics	Univariate Analysis			Multivariate Analysis		
	HR	95% CI	*p*-Value	HR	95% CI	*p*-Value
Proteinuria	0.637	0.423–0.960	0.031	0.592	0.391–0.896	0.013
Anemia	2.505	1.521–4.125	<0.001	2.599	1.569–4.306	<0.001
Age ^1^	0.764	0.510–1.145	0.192	-	-	-
Cardiovascular comorbidities	1.263	0.838–1.904	0.264	-	-	-
Diabetes	1.485	0.784–2.813	0.225	2.264	1.171–4.376	0.015
Staging at diagnosis ^2^	0.495	0.310–0.791	0.003	0.454	0.282–0.731	0.001
Number of metastatic sites ^3^	0.660	0.439–0.993	0.046	0.558	0.369–0.846	0.006
Tumor location ^4^	0.944	0.760–1.172	0.601	-	-	-

^1^ Less or more than 65 years; ^2^ Staging at diagnosis: metastatic versus non-metastatic; ^3^ One or more than one metastatic site; ^4^ Left versus right.

## Data Availability

The datasets generated and analyzed during the current study are available from the corresponding author and can be shared with the journal for review if needed.

## Supplementary Materials

The following supporting information can be downloaded at: https://www.mdpi.com/article/10.3390/curroncol29060319/s1, Table S1: SPM.
